# Supplementary material for: Attitudes towards the Tick-Borne Encephalitis Vaccine among Children’s Guardians: A Cross-Sectional Survey Study in Poland
Source: Vaccines (Basel). 2024 Aug 15;12(8):918. doi: 10.3390/vaccines12080918 (PMC11360253; doi:10.3390/vaccines12080918)
Supplement: Supplementary file 1 [file vaccines-12-00918-s001.zip › vaccines-3123308-supplementary.pdf]

Table S1. Overall parental attitudes toward non-mandatory childhood vaccinations and by the willingness to vaccinate children against TBE.

Characteristic	N	Overall sample	Willingness to vaccinate children against TBE		p[b]
			yes, n = 872[a]	no, n = 2158[a]	
Q5. Against which of the following diseases, which are not included in the prescribed vaccination schedule, do you want to have your child vaccinated?					
TBE	3,030	872 (28.78%)	-	-	**-**
Chickenpox	3,029	1,112 (36.71%)	557 (63.88%)	555 (25.73%)	**< 0.001**
Influenza	3,029	521 (17.20%)	322 (36.93%)	199 (9.23%)	**< 0.001**
Meningococcus	3,030	1,175 (38.78%)	590 (67.66%)	585 (27.11%)	**< 0.001**
Hepatitis A virus	3,030	606 (20.00%)	407 (46.67%)	199 (9.22%)	**< 0.001**
HPV	3,030	700 (23.10%)	409 (46.90%)	291 (13.48%)	**< 0.001**
None of the above	3,030	1,202 (39.67%)	2 (0.23%)	1,200 (55.61%)	**< 0.001**

[a] n (%)
[b] Pearson's Chi-squared test

Table S2. Overall comparative analysis of information sources on vaccinations and by the willingness to vaccinate children against TBE.

Characteristic	N	Overall sample	Willingness to vaccinate children against TBE		p[b]
			yes, n = 872[a]	no, n = 2,158[a]	
Q.8 Where do you get your knowledge about vaccinations from?					
Doctor	3,029	2,068 (68.27%)	634 (72.71%)	1,434 (66.48%)	**0.001**
Pharmacist	3,030	255 (8.42%)	85 (9.75%)	170 (7.88%)	0.093
Nurse	3,030	646 (21.32%)	197 (22.59%)	449 (20.81%)	0.277
Friends	3,030	287 (9.47%)	80 (9.17%)	207 (9.59%)	0.722
Popular science books and guides	3,030	722 (23.83%)	143 (16.40%)	579 (26.83%)	**< 0.001**
CDC & WHO	3,030	1,285 (42.41%)	425 (48.74%)	860 (39.85%)	**< 0.001**
TV	3,030	178 (5.87%)	70 (8.03%)	108 (5.00%)	**0.001**
Internet	3,030	1,952 (64.42%)	525 (60.21%)	1,427 (66.13%)	**0.002**
Social media	3,030	610 (20.13%)	167 (19.15%)	443 (20.53%)	0.392
Posters and flyers	3,029	458 (15.12%)	151 (17.34%)	307 (14.23%)	**0.031**
Non-medical journals	3,029	150 (4.95%)	24 (2.76%)	126 (5.84%)	**< 0.001**

[a] n (%)
[b] Pearson's Chi-squared test

Table S3. Perception of infectious diseases as dangerous*.

Characteristic (infectious disease)	N	Overall sample	Willingness to vaccinate children against TBE		p^b
			yes, n = 872[a]	no, n = 2,158[a]	
Chickenpox	3,026	1,690 (55.85%)	651 (74.91%)	1,039 (48.17%)	**< 0.001**
Influenza	3,026	2,136 (70.59%)	730 (84.00%)	1,406 (65.18%)	**< 0.001**
TBE	3,027	2,905 (95.97%)	860 (98.62%)	2,045 (94.90%)	**< 0.001**
Invasive meningococcal disease	3,017	2,826 (93.67%)	852 (98.04%)	1,974 (91.90%)	**< 0.001**
Hepatitis A virus	3,024	2,790 (92.26%)	842 (96.78%)	1,948 (90.44%)	**< 0.001**
HPV	3,008	2,650 (88.10%)	816 (94.99%)	1,834 (85.34%)	**< 0.001**
SARS-CoV-2	1,817	1,420 (78.15%)	492 (90.61%)	928 (72.84%)	**< 0.001**

[a] n (%)
[b] Pearson's Chi-squared test

*From rather to very dangerous.

Table S4. Perception vaccine safety for infectious diseases as safe*.

Characteristic (vaccine)	N	Overall sample	Willingness to vaccinate children against TBE		p[b]
			yes, n = 872[a]	no, n = 2,158[a]	
Chickenpox vaccine	3,013	1,967 (65.28%)	758 (86.93%)	1,209 (56.02%)	**< 0.001**
Influenza vaccine	3,015	1,661 (55.09%)	666 (76.38%)	995 (46.11%)	**< 0.001**
TBE vaccine	3,010	1,835 (60.96%)	750 (86.00%)	1,085 (50.28%)	**< 0.001**
Meningococcal vaccine	3,006	1,768 (58.82%)	714 (81.88%)	1,054 (48.84%)	**< 0.001**
Hepatitis A vaccine	3,009	1,875 (62.31%)	742 (85.09%)	1,133 (52.50%)	**< 0.001**
HPV vaccine	3,007	1,746 (58.06%)	707 (81.08%)	1,039 (48.15%)	**< 0.001**
COVID-19 mRNA vaccine	1,804	709 (39.30%)	302 (34.63%)	407 (18.86%)	**< 0.001**

[a] n (%)
[b] Pearson's Chi-squared test

* From rather to very safe.

Text-file S5. Complete questionnaire filled out by participants.

1. Do you think children should be vaccinated?
☐ 1. Yes
☐ 2. No
☐ 3. I don't know.

2. What do you think is more dangerous?
☐ 1. Vaccination of children
☐ 2. No vaccination
☐ 3. I don't know.

3. Which methods of boosting immunity do you think are effective (you can choose more than one)?
☐ 1. Breastfeeding
☐ 2. Vitamin supplementation
☐ 3. Probiotic supplementation
☐ 4. Bathing in ice-cold water
☐ 5. Using saunas
☐ 6. Using solariums
☐ 7. Daily physical activity
☐ 8. Balanced healthy diet
☐ 9. Consumption of herbs and natural products, such as coneflower, ginseng, aloe vera, ginger, turmeric, garlic, raspberry juice, cod liver oil
☐ 10. Other (what?):

4. Do you think natural methods of boosting immunity are more effective in preventing infections than vaccination?
☐ 1. Yes
☐ 2. No
☐ 3. I don't know.

5. Against which of the following diseases, that are not included in the mandatory vaccination schedule, do you plan on vaccinating your child? (you can choose more than one)
☐ 1. Chickenpox
☐ 2. Influenza
☐ 3. COVID
☐ 4. Tick-borne encephalitis
☐ 5. Meningococcal disease
☐ 6. Hepatitis A (foodborne hepatitis)
☐ 7. Human papillomavirus (HPV)
☐ 8. None of the above

6. The use of highly combined vaccines (e.g., 6-in-1) makes it possible to reduce the number of necessary pricks during one visit, which is associated with less pain for the child. These vaccines are widely used in infants in most European countries, but in Poland their cost is covered by the parents. With the possibility of free access to these highly combined vaccines, would you choose to give them to your child?
☐ 1. Yes
☐ 2. No
☐ 3. I don't know.

7. What problems have you encountered when talking to your doctor about immunization?
☐ 1. I have not encountered any problems.
☐ 2. The doctor does not have time to talk to the patient
☐ 3. The doctor spends enough time with each patient, but doesn't want to talk about vaccinations
☐ 4. The doctor has time and wants to talk, but during the conversation (s)he uses difficult terminology, making me not fully understand what (s)he is talking about.
☐ 5. Other (what?):

8. Where do you get your knowledge about vaccination?
☐ 1. Doctor
☐ 2. Pharmacist
☐ 3. Nurse
☐ 4. Friends without medical training
☐ 5. Popular science books and guides
☐ 6. Professional sources of medical knowledge (e.g. Practical Medicine, CDC, WHO, medical textbooks, etc.)
☐ 7. Television
☐ 8. Internet (various vaccination sites)
☐ 9. Social media (Facebook, Twitter)
☐ 10. Posters and flyers
☐ 11. Non-medical magazines
☐ 12. Other sources (which ones?):

9. In your opinion, can vaccinations cause autism?
☐ 1. Yes
☐ 2. No
☐ 3. I don't know.

10. How much of a health threat do you consider the following infectious diseases to be?
Mark a number on a scale of 1 to 5, where 1 means that the infectious disease is not a health threat at all and is harmless, and 5 means that the disease is very dangerous.

Infectious Disease	Not dangerous				Very dangerous
Chickenpox	☐ 1	☐ 2	☐ 3	☐ 4	☐ 5
Influenza	☐ 1	☐ 2	☐ 3	☐ 4	☐ 5
Infection with SARS-CoV-2 virus causing COVID-19	☐ 1	☐ 2	☐ 3	☐ 4	☐ 5
Tick-borne encephalitis	☐ 1	☐ 2	☐ 3	☐ 4	☐ 5
Meningococcal disease	☐ 1	☐ 2	☐ 3	☐ 4	☐ 5
Hepatitis A (HepA)	☐ 1	☐ 2	☐ 3	☐ 4	☐ 5
Human papillomavirus (HPV)	☐ 1	☐ 2	☐ 3	☐ 4	☐ 5

11. How safe do you think the following vaccines are?
Rate the safety of the vaccines on a scale of 1 to 5, where 1 means that you think the vaccine is not safe and often causes serious vaccine reactions, and 5 that the vaccine is very safe.

Vaccine	Not safe				Very safe
Chickenpox vaccine	☐ 1	☐ 2	☐ 3	☐ 4	☐ 5
Influenza vaccine	☐ 1	☐ 2	☐ 3	☐ 4	☐ 5
COVID-19 mRNA vaccine	☐ 1	☐ 2	☐ 3	☐ 4	☐ 5
Tick-borne encephalitis (TBE) vaccine	☐ 1	☐ 2	☐ 3	☐ 4	☐ 5
Meningococcal vaccine	☐ 1	☐ 2	☐ 3	☐ 4	☐ 5
Hepatitis A (HepA) vaccine	☐ 1	☐ 2	☐ 3	☐ 4	☐ 5
Human papillomavirus (HPV) vaccine	☐ 1	☐ 2	☐ 3	☐ 4	☐ 5

12. Is there anything you would like to ask your doctor regarding vaccinations? (write down your question here)
☐ ....
☐ I have no questions.

13. Gender
☐ 1. Female
☐ 2. Male

14. Age
☐ 1. 10-19 years
☐ 2. 20-29 years
☐ 3. 30-39 years
☐ 4. 40-49 years
☐ 5. 50-59 years
☐ 6. 60-69 years
☐ 7. 70 years and over

15. Education
☐ 1. Primary
☐ 2. Secondary
☐ 3. Vocational
☐ 4. Higher

16. Place of residence
☐ 1. Rural

☐ 2. City with up to 30 000 residents
☐ 3. City with up to 300 000 residents
☐ 4. City with over 300 000 residents

17. Do you have children? Number of children: ....
(Categorized as:
☐ 1. Participant has 1 child
☐ 2. Participant has 2 children
☐ 3. Participant has 3 children
☐ 4. Participant has 4 children and more)

18. In which voivodeship do you currently reside?
....
